# A quantitative proteomic analysis of cofilin phosphorylation in myeloid cells and its modulation using the LIM kinase inhibitor Pyr1

**DOI:** 10.1371/journal.pone.0208979

**Published:** 2018-12-14

**Authors:** Renaud Prudent, Nathalie Demoncheaux, Hélène Diemer, Véronique Collin-Faure, Reuben Kapur, Fabrice Paublant, Laurence Lafanechère, Sarah Cianférani, Thierry Rabilloud

**Affiliations:** 1 CELLIPSE SAS, Grenoble, France; 2 Laboratoire de Spectrométrie de Masse BioOrganique (LSMBO), Université de Strasbourg, CNRS, IPHC UMR 7178, Strasbourg, France; 3 Chemistry and Biology of Metals, Univ. Grenoble Alpes, CNRS UMR5249, CEA, BIG-LCBM, Grenoble, France; 4 Department of Pediatrics, Herman B Wells Center for Pediatric Research, Indiana University School of Medicine, Indianapolis, United States of America; 5 Institute for Advanced Biosciences, Univ. Grenoble Alpes, CNRS UMR 5309, INSERM U1209; Grenoble, France; Centre National de la Recherche Scientifique, FRANCE

## Abstract

LIM kinases are located at a strategic crossroad, downstream of several signaling pathways and upstream of effectors such as microtubules and the actin cytoskeleton. Cofilin is the only LIM kinases substrate that is well described to date, and its phosphorylation on serine 3 by LIM kinases controls cofilin actin-severing activity. Consequently, LIM kinases inhibition leads to actin cytoskeleton disorganization and blockade of cell motility, which makes this strategy attractive in anticancer treatments. LIMK has also been reported to be involved in pathways that are deregulated in hematologic malignancies, with little information regarding cofilin phosphorylation status. We have used proteomic approaches to investigate quantitatively and in detail the phosphorylation status of cofilin in myeloid tumor cell lines of murine and human origin. Our results show that under standard conditions, only a small fraction (10 to 30% depending on the cell line) of cofilin is phosphorylated (including serine 3 phosphorylation). In addition, after a pharmacological inhibition of LIM kinases, a residual cofilin phosphorylation is observed on serine 3. Interestingly, this 2D gel based proteomic study identified new phosphorylation sites on cofilin, such as threonine 63, tyrosine 82 and serine 108.

## Introduction

Targeting cytoskeletal components, such as actin or tubulin has been shown to inhibit the invasive and metastatic behavior of cancer cells [1, 2]. Pharmacological agents targeting the cytoskeleton are however toxic for many proliferating normal cells as well as peripheral neurons, inducing severe adverse effects. Novel alternative pharmacological strategies aim at targeting cytoskeleton-regulating proteins, which are selectively deregulated during cancer progression. LIM kinases (LIMK), a family of two highly related members, LIMK1 and LIMK2, which control both actin and microtubule dynamics [3] and are overexpressed in many invasive cancers, are an attractive target [4, 5].

The initially- and most extensively- described substrates of LIMK are members of the actin-depolymerizing factor (ADF)/cofilin family of actin-binding proteins (Cofilin1, cofilin 2 and ADF), which control actin cytoskeleton dynamics [6].

LIM kinases phosphorylate cofilin on serine 3 [7], which leads to cofilin inactivation, resulting in reorganisation of the actin cytoskeleton. In adherent cells LIMK increases stress fibre and focal adhesion formation by reducing actin depolymerisation. LIMK-initiated changes in F-actin severing also leads to reduced barbed end formation and leading edge protrusions.

An overexpression of LIMK is observed in many invasive cancers, which could be associated with increased EMT [8] and motility acquisition by metastatic cells.

Upon LIMK inhibition, microtubules are stabilized and actin filaments are disorganized [3, 9,10]. Western blotting with phospho-S3 cofilin-specific antibodies, show in most cases a strong decrease of S3 phosphorylation [9–14]. Recent findings indicate that pharmacological inhibition of LIMK has a strong effect on the growth and invasive behavior of breast cancer cells in 3D cell models [15] and in animal tumor models [16]. Such a treatment was well-tolerated and showed efficacy even on taxane resistant tumors [16].

Recent data also indicate that inhibition of LIMK may also be of interest in hematologic malignancies, where overexpression of a signaling pathway involving LIMK is observed [17, 18]. Several reports suggest that targeting LIMK [19] in leukemia may be of therapeutic value. Especially, it has been evidenced that inhibiting LIMK exerts an anti-leukemic activity in murine model of leukemia [10].

With the ultimate goal of better understanding the role of LIMK and the dynamic regulation of the cytoskeleton in the functions of these non-epithelial cell types, we first analyzed in depth the phosphorylation of cofilin and studied how it was impacted by inhibition of LIMK using the well-described Pyr1 inhibitor [3]. Since the WB technique is only grossly quantitative [20–23], we analyzed the degree of cofilin phosphorylation by phospho-proteomics.

This approach made it possible to demonstrate that in these cell types, under our experimental conditions, a residual phosphorylation of cofilin can be o bserved on serine 3 after pharmacological inhibition of LIMK. In addition, we have identified new phosphorylated sites. Since Ser 3 cofilin is the main site of actin severing regulation and considering the phylogenic conservation of these new sites, it is likely that multiples regulations of cofilin are yet to be discovered.

## Material and methods

### Chemical reagent

Pyr1 was synthetized as described in [10].To maximize its effect it was used at 25 μM, its maximal soluble concentration in most cell culture media. Pyr-1 was prepared as a concentrated solution in DMSO, and an amount of DMSO equal to the one that is contained in the 25 M Pyr1 solution was added to the control cultures.

### Cell lines and cell culture

MV4-11 and Kasumi-1 cell lines were originally purchased from the American Type Culture Collection (ATCC). MV4-11and Kasumi-1 cells were cultured in RPMI 1640 10% (v/v) fetal bovine serum FBS supplemented with 100 U/ml penicillin, 0.1mg/ml streptomycin (PAN Biotech P06-07100) and 2mM Glutamine (Sigma-Aldrich 59202C). Murine IL-3-dependent myeloid cell line 32D cells bearing MIEG3 vector, KIT WT or KITD814V mutant were cultured in medium containing IMDM supplemented with 10% FBS and murine IL-3 (10 ng/ml). Cells were maintained at 37°C with 5% CO_2_. For treatment with Pyr1, cells were treated for 2 hours with 25 μM Pyr1 or with DMSO.

For the cell proliferation assay, cells were seeded in 96 wells microplates at 5,000 cells per well (MV 4–11, 32D-WT and 32D-D814V cell lines) or at 20,000 cells per well (Kasumi-1 cell line). Compounds (or equivalent amounts of DMSO) were then added and cells were allowed to grow for 48 hours (MV4-11, 32D-WT and 32D-D814V cell lines) or 96 hours (Kasumi-1cell line). Proliferation was evaluated using PrestoBlue assay according to manufacturer recommendations. Results are expressed as GI50 (Growth Inhibition 50%), TGI (Total Growth Inhibition) and LD50 (Letal Dose 50%) in comparison to DMSO controls.

### Proteomics

The 2D gel based proteomic experiments were essentially carried out as previously described [24]. However, detailed material and methods are provided for the sake of paper consistency and for phosphopeptide identification and quantification.

#### Sample preparation

The cells were collected, and then washed three times in PBS. The cells were then washed once in TSE buffer (10 mM Tris-HCl pH 7.5, 0.25M sucrose, 1mM EDTA), and the volume of the cell pellet was estimated. The pellet was resuspended in its own volume of TSE buffer. Then 4 volumes (respective to the cell suspension just prepared) of concentrated lysis buffer (8.75 M urea, 2.5 M thiourea, 5% (w/v) CHAPS, 6.25 mM TCEP-HCl, 12.5 mM spermine base, 25 mM HCl) were added and the solution was let to extract at room temperature for 1 hour. The nucleic acids were then pelleted by centrifugation (15,000 g at room temperature for 15 minutes), and the protein concentration in the supernatant was determined by a dye-binding assay [25]. Carrier ampholytes (Pharmalytes pH 3–10) were added to a final concentration of 0.4% (w/v), and the samples were kept frozen at -20°C until use.

#### Isoelectric focusing

Home-made 160 mm long 4–8 linear pH gradient gels [26] cast according to published procedures [27] or commercial non linear 3–10, 18 cm long pH gradients (Serva) were used. In the former case, four mm-wide strips were cut. The strips were rehydrated overnight with the sample, diluted in a final volume of 0.6 ml of rehydration solution (7M urea, 2M thiourea, 4% (w/v) CHAPS, 0.4% carrier ampholytes (Pharmalytes 3–10) and 100mM dithiodiethanol [28] for the 4 mm-wide strips and in 0.35 ml of rehydration solution for the commercial strips.

The strips were then placed in a Multiphor plate (GE Healthcare), and IEF was carried out with the following electrical parameters: 100V for 1 hour, then 300V for 3 hours, then 1000V for 1 hour, then 3400 V up to 60–70 kVh. After IEF, the gels were equilibrated for 20 minutes in 125mM Tris, 100mM HCl, 2.5% SDS, 30% (v/v) glycerol and 6M urea [29]. They were then transferred on top of the SDS gels and sealed in place with 1% agarose dissolved in Tris 125mM, HCl 100mM, SDS 0.4% and 0.005% (w/v) bromophenol blue.

#### SDS electrophoresis and protein detection

Ten percent gels (160x200x1.5 mm) were used for protein separation. The Tris taurine buffer system [30] was used and operated at a ionic strength of 0.1 and a pH of 7.9. The final gel composition was thus 180mM Tris, 100 mM HCl, 10% (w/v) acrylamide, 0.27% bisacrylamide. The upper electrode buffer was 50 mM Tris, 200 mM taurine, 0.1% SDS. The lower electrode buffer was 50 mM Tris, 200 mM glycine, 0.1% SDS. The gels were run at 25V for 1hour, then 12.5W per gel until the dye front has reached the bottom of the gel. Detection was carried out by a tetrathionate silver staining [31].

#### Blotting

When blotting was performed, 2D gels were used immediately after the second dimension migration. Proteins were transferred on PVDF membranes using the semidry blotting system of Kyhse-Andersen,[32] with some of the modifications introduced by Laurière [33]. Briefly, the anode buffers, including the PVDF-impregnating buffer contained 20% ethanol (v/v), while the cathode buffer contained 0.1% SDS (w/v). The electrophoretic transfer proceeded at 0.8 mA/cm2 of membrane. After transfer, the blot was first stained overnight with india ink, and then submitted to immunodetection [34], with all steps performed at room temperature. To this purpose, the PVDF membrane (Merck-Millipore Immobilon P IPVH00010) is blocked with Tris Buffered Saline, pH 7.4 (TBS) with 0.1% Tween 20 and 5% BSA for 1h under agitation. The membrane was rinsed 3 times 10min under agitation with TBS, 0.1% Tween-20 (TBST) and then 1hour with anti-phospho-Ser3-cofilin (Cell Signaling Technology #3313, 1/1000 dilution) antibody or anti-cofilin (Cell Signaling Technology #3312, 1/1000 dilution) antibody diluted in TBS, 0.1% Tween 20 and 1% BSA. The membrane is then rinsed 3 times 10min under agitation in TBST and incubated for 1 hour with anti-rabbit secondary antibody, horseradish peroxidase conjugated (Jackson Immunoresearch #711-036-152) under agitation. After three washes (10min each) in TBST, the detection of total or phosphorylated cofilin was performed using chemiluminescence kit ECL Plus (GE Healthcare RPN2132). The signal was acquired on a C-Digit model 3600 (Licor).

#### Image analysis

The gels were scanned after silver staining on a flatbed scanner (Epson perfection V750), using a 16 bits grayscale image acquisition. The gel images were then analyzed using the Delta 2D software (v 4.1). The spots intensities were normalized by the software as the fraction (in percent) of the sum of all detected spots. This allowed the quantitative comparison of the various cofilin spots in all gels analyzed.

For determining the effects of LIMK inhibition on cofilin phosphorylation, statistical tests were used. As different culture batches can show quantitatively different 2D gel profiles, paired (by culture batch) and unpaired (over the complete replicate series) T-tests were used.

#### Mass spectrometry

The spots selected for identification were excised from silver-stained gels and destained with ferricyanide/thiosulfate on the same day as silver staining in order to improve the efficiency of the identification process [35] [36]. In gel digestion was performed with an automated protein digestion system, MassPrep Station (Waters, Milford, USA). The gel plugs were washed twice with 50 μL of 25 mM ammonium hydrogen carbonate (NH_4_HCO_3_) and 50 μL of acetonitrile. The cysteine residues were reduced by 50 μL of 10 mM dithiothreitol at 57°C and alkylated by 50 μL of 55 mM iodoacetamide. After dehydration with acetonitrile, the proteins were cleaved in gel with 10 μL of 7 ng/μL of modified porcine trypsin (Promega, Madison, WI, USA) in 25 mM NH_4_HCO_3_. The digestion was performed overnight at room temperature. The generated peptides were extracted with 40 μL of 60% acetonitrile in 0.1% formic acid. Acetonitrile was evaporated under vacuum before nanoLC-MS/MS analysis.

NanoLC-MS/MS analysis was performed using a nanoACQUITY Ultra-High-Performance-LC (Waters Corporation, Milford, USA) coupled to the TripleTOF 5600 (Sciex, Ontario, Canada).

The nanoLC system was composed of ACQUITY UPLC CSH130 C18 column (250 mm x 75 μm with a 1.7 μm particle size, Waters Corporation, Milford, USA) and a Symmetry C18 precolumn (20 mm × 180 μm with a 5 μm particle size, Waters Corporation, Milford, USA). The solvent system consisted of 0.1% formic acid in water (solvent A) and 0.1% formic acid in acetonitrile (solvent B). 4 μL of sample were loaded into the enrichment column during 3 min at 5 μL/min with 99% of solvent A and 1% of solvent B. Elution of the peptides was performed at a flow rate of 300 nL/min with a 8–35% linear gradient of solvent B in 9 minutes.

The TripleTOF 5600 (Sciex, Ontario, Canada) was operated in positive mode, with the following settings: ion spray voltage floating (ISVF) 2300 V, curtain gas (CUR) 10, interface heater temperature (IHT) 150, ion source gas 1 (GS1) 2, declustering potential (DP) 80 V. Data-Dependent Acquisition was performed using the Information-Dependent Acquisition (IDA) mode with Top 10 MS/MS scans. The MS scan had an accumulation time of 250 ms on m/z [400;1250] range and the MS/MS scans 100 ms on m/z [150;1800] range in high sensitivity mode. Switching criteria were set to ions with charge state of 2+ - 4+ and an abundance threshold of more than 500 counts, exclusion time was set at 4 s. IDA rolling collision energy script was used for automatically adapting the CE. Mass calibration of the analyzer was achieved using peptides from digested BSA. The complete system was fully controlled by AnalystTF 1.7 (Sciex). Raw data collected were processed and converted with MSDataConverter in .mgf peak list format.

For protein identification, the MS/MS data were interpreted using a local Mascot server with MASCOT 2.5.1 algorithm (Matrix Science, London, UK) against UniProtKB/SwissProt (version 2016_05, 551,193 sequences). The research was carried out without taxonomy restrictions. Spectra were searched with a mass tolerance of 15 ppm for MS and 0.05 Da for MS/MS data, allowing a maximum of one trypsin missed cleavage. Carbamidomethylation of cysteine residues, oxidation of methionine residues, acetylation of protein n-terminus and phosphorylation of serine, threonine and tyrosine residues were specified as variable modifications. Protein identifications were validated with at least two peptides with Mascot ion score above 30. The mass spectrometry data have been deposited in PRIDE [37].

For cofilin phosphopeptide quantitative analyses, XIC signals of the all peptides identified in the same spot were extracted using the Skyline software (version 4.1.0.11796). Total areas, corresponding to the sum of the 3 extracted isotopes areas, were used for quantitative analysis, as described by Schilling et al [38].

To compensate for differences in MS ionization efficiencies between phosphorylated and non-phosphorylated peptides, we normalized our data in the following manner: the quantification of a given cofilin phosphopeptide in spot III was estimated as the ratio of intensity of the signal of the targeted cofilin phosphopeptide divided by the sum of the intensities of all detected peptides (phosphorylated + nonphosphorylated) in spot III. Although this ratio per se does not provide an absolute value of the abundance of the phosphopeptide of interest, the ratio of the ratios obtained for the same cofilin phosphopeptide in two different experimental conditions (e.g. control vs Pyr1-treated) was used as an indicator of the relative abundance of the phosphopeptide of interest at constant protein amount (same protein background in the spots to be compared) and is independent from differences in ionization efficiencies between peptides.

## Results

### Determination of Pyr1 effects on cells

The effects of Pyr1 on the cell lines used were first investigated. Using a treatment of 48 hours, the growth inhibitory and lethal doses were determined. The results, displayed in **Table 1**, show a moderate toxicity and an efficient growth inhibition of the four cell lines tested. We also verified that even at 25 μM, the two-hours treatment used to analyze the effects of LIMK inhibition did not result in any toxicity.

**Table 1:** Effective Pyr1 concentrations on the different cell lines for a 48 hours treatment.

cell line	GI 50 (μM)	LD 50 (μM)
MV4-11 DSMZ	1.4	17.5
Kasumi-1	1.2	10.3
32D c-KIT WT	0.11	14.5
32D c-KIT D814V	0.42	12.9

GI 50: Dose producing a 50% inhibition of cell growth over the treatment time (48 hoursLD 50: Dose producing 50% cell death over the treatment time (48 hours)

### Assignment of the cofilin spots on the 2D gel patterns

**Fig 1:**
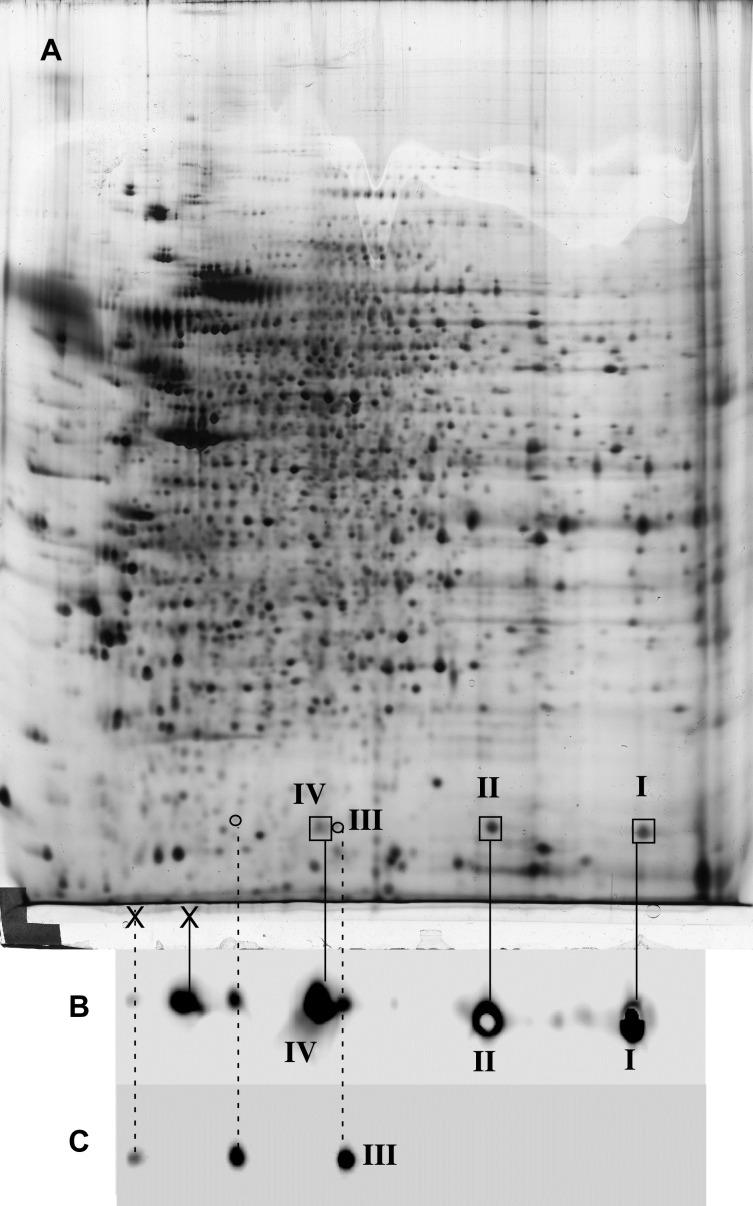
Alignment of cofilin spots. The process of alignment and detection of cofilin and phosphocofilin spots has been carried out on the 32D c-kit WT cell line. Proteins were separated on a commercial non linear 3–10 pH gradient. A: silver stained pattern of total proteins. B: western blotting of cofilin spots (I, II, III,IV) with a polyclonal anti-cofilin antibody. C: western blotting of phospho-cofilin spot (III) with an anti phospho S3 peptide antibody The spots labelled with an X correspond to spots that are detected by the antibodies but that could not be confirmed by mass spectrometry.

The assignment of the cofilin spots on the 2D gel patterns was initiated by protein blotting and confirmed by mass spectrometry. After transfer on PVDF, the membranes were probed successively with an anti phospho-S3 antibody and with a polyclonal anti-cofilin antibody. The results, displayed on **Fig 1**(with more details shown on S1 Fig) show that only one acidic spot (Spot III, Fig 1A–1C) was phosphorylated on S3, while three other major spots (Spots I, II and IV Fig 1A and 1B) were detected by blotting and confirmed by mass spectrometry. The basic spots (I and II) made indeed the bulk of the cofilin pool. The identity of the spots was confirmed by mass spectrometry for both human and murine cofilin (**Table 2)**. As shown in S1 Fig, extensive spot identification by mass spectrometry was carried out to assign the more acidic spots detected by the antibodies on the blots. We could however not confirm the identity of these acidic spots as cofilin. These spots were thus considered as immunoblotting artefacts.

**Table 2:** Spot identification by mass spectrometryT.

Cell line	spot	Protein name	Protein accession numbers	Protein molecular weight (Da)	Exclusive unique peptide count	Percentage sequence coverage
Kasumi	IV	Cofilin-1 OS = Homo sapiens	P23528	18 503,2	12	51%
	III	Cofilin-1 OS = Homo sapiens	P23528	18 503,2	14	54%
	II	Cofilin-1 OS = Homo sapiens	P23528	18 503,2	10	54%
	I	Cofilin-1 OS = Homo sapiens	P23528	18 503,2	12	61%
32D	IV	Cofilin-1 OS = Mus musculus	P18760	18 560,2	12	65%
	III	Cofilin-1 OS = Mus musculus	P18760	18 560,2	4	27%
	II	Cofilin-1 OS = Mus musculus	P18760	18 560,2	19	78%
	I	Cofilin-1 OS = Mus musculus	P18760	18 560,2	23	84%
	X	ARP 2/3 complex subunit 5-like protein OS = Mus musculus	Q9D898	16 941,4	3	20%

The identity of the cofilin spots for the MV 4–11 and 32D-D814V cell lines was inferred by spot matching on a cell line from the same species, i.e. on the Kasumi cell line for MV 4–11 and on the 32D-WT for the 32D-D814V cell line

The silver-stained gels were then used to quantify the various forms on several myeloid cell lines, two from human origin and the 32D cell line from murine origin, with or without a mutation (D814V) of c-kit proto-oncogene. Such a mutation is known to induce imatinib-resistant aggressive leukemia and myeloproliferative disorders [39] [40] through a ROCK-LIMK signaling pathway [18].

**Fig 2:**
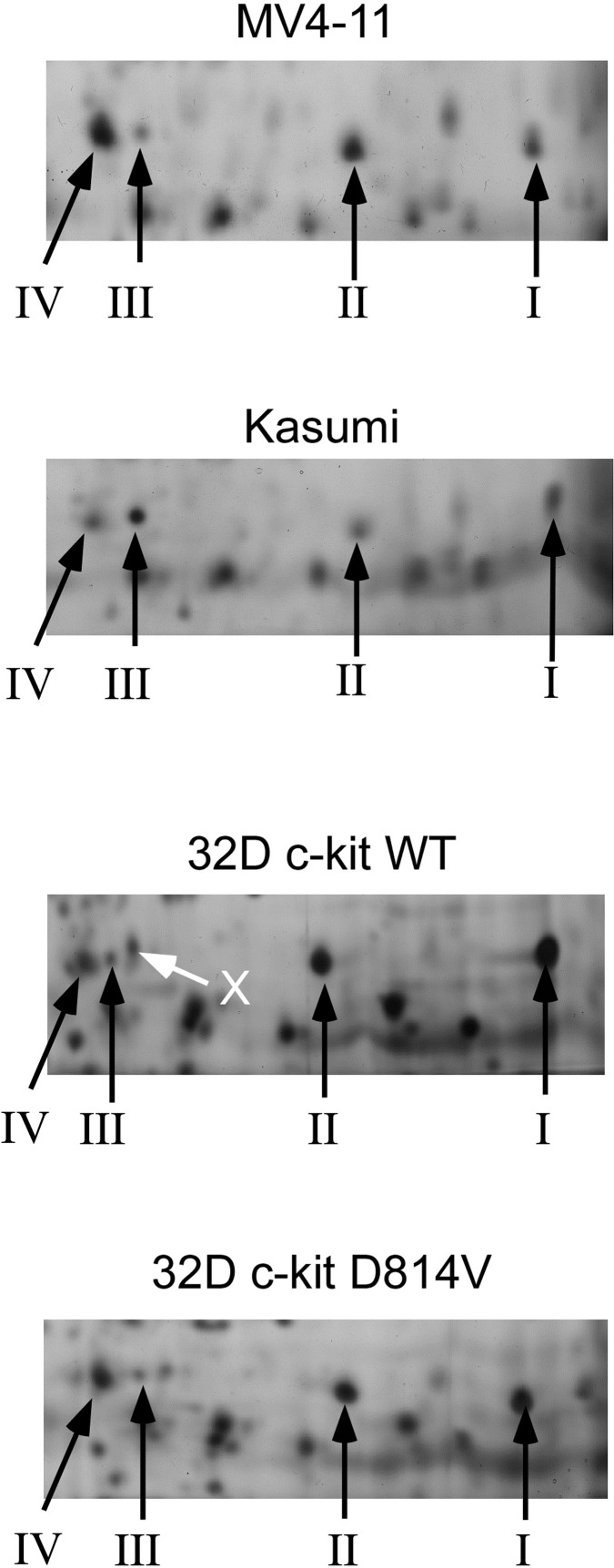
Cofilin spots profiles of the four cell lines tested. The areas containing the cofilin spots (I to IV) have been selected for the sake of the clarity of the figure. The protein separation used the same pH gradient as the one used in Fig 1. Note the close vicinity of other protein spots (X) with the phosphocofilin spot (III) in the murine cell lines. In these images, proteins were detected by silver staining.

As shown on **Fig 2**, the phosphorylated cofilin spot (Spot III) represented a minor percentage of the total cofillin in all the cell lines tested, and varied from one cell line to another.

In the wide pH gradients used for the cofilin spots assignment, the area of the phosphorylated cofilin spot is crowded. This may lead to quantitative errors when a more precise appraisal of the phosphorylated cofilin spot is required.

### LIMK inhibition induces a partial decrease of the phosphorylated cofilin spot

**Fig 3:**
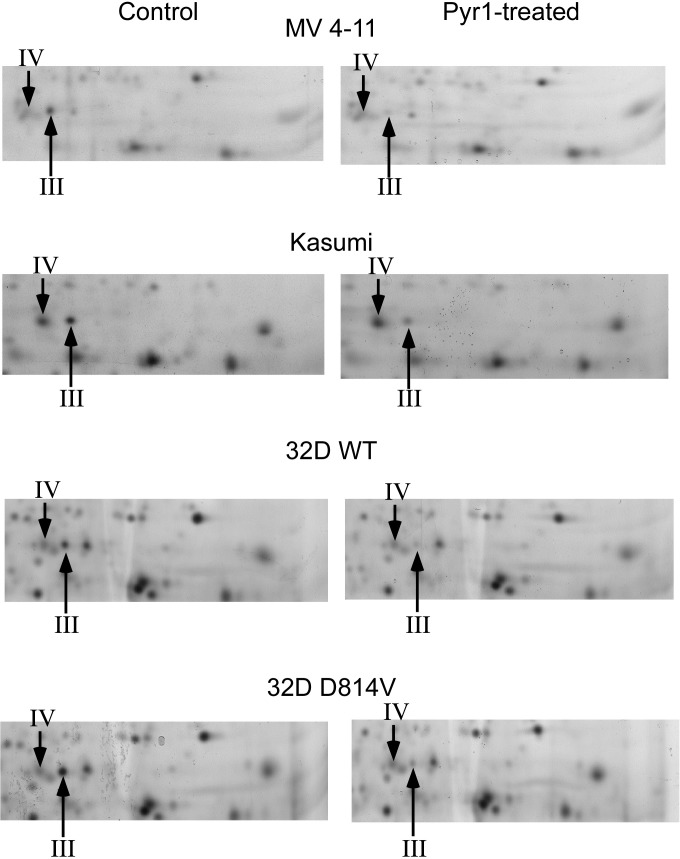
Phosphocofilin intensity decrease upon LIMK inhibition. To reduce the spot crowding around the phosphocofilin spot, a linear 4–8 pH gradient was used. This does not allow the visualization of the basic cofilin spot (spot I), and brings the median spot (II) to the border of the gel, rendering its quantitative measurement unreliable. By contrast, the phosphocofilin spot (III) is more separated from neighboring spots and can be more precisely quantitatively measured. It is also worth noting that spot IV, which is not detected by the anti phospho S3 peptide antibody, does not respond to Pyr-1 treatment. In these images, proteins were detected by silver staining.

To quantitatively investigate the changes of cofilin phosphorylation brought through LIMK inhibition by Pyr1, we used linear 4–8 pH gradients which allowed a more precise resolution of the phosphorylated cofilin spot area than the wide pH gradients used previously for the cofilin spots assignment. Typical silver-stained 2D images of the cofilin regions are shown on **Fig 3**and the detailed quantitative results are displayed in **Table 3**. An important decrease of the phosphorylated cofilin spot intensity upon Pyr1 treatment was seen in all cases. The decrease was however incomplete, ranging from 1.5fold (MV4-11) to 3fold (32D-D814V)

**Table 3:** quantitative measurements of the phosphocofilin spots. The data for spot I abundance are expressed in percentage of the sum of all spots measured on the silver-stained gels (see DOI: 10.5061/dryad.4n5929c for the complete gel images)The proteomic experiments have been carried out on four independent series for the human cell lines (1 to 4 in the table below) and on three independent series for the murine cell lines (1 to 3 in the table below).

		Spot I	intensity		paired	unpaired
cell line	experimental	ctrl	pyr1	ratio	T-test	T-test
	series					
MV4-11	1	0.3455	0.2257	0.6533	0.024	0.141
	2	0.2493	0.1444	0.5791		
	3	0.1683	0.1412	0.8389		
	4	0.1774	0.0796	0.4487		
	mean	0.2351	0.1477			
	ratio of means		0.6283			
	mean of individual ratios					
Kasumi-1	1	0.1392	0.0856	0.6148	0.026	0.026
	2	0.3041	0.1099	0.3612		
	3	0.2562	0.1523	0.5942		
	4	0.2423	0.0789	0.3258		
	mean	0.2355	0.1067			
	ratio of means		0.4529			
	mean of individual ratios		0.4740			
32D	1	0.0881	0.0546	0.6195	0.080	0.121
c-kit WT	2	0.0787	0.0253	0.3211		
	3	0.0420	0.02473	0.5891		
	mean	0.0696	0.0348			
	ratio of means		0.5009			
	mean of individual ratios		0.5099			
32D	1	0.1364	0.0584	0.4279	0.0027	0.0054
c-kit D814V	2	0.1311	0.0380	0.2896		
	3	0.1062	0.0171	0.1606		
	mean	0.1246	0.0378			
	ratio of means		0.3034			
	mean of individual ratios		0.2927			

Notes: the variability of some cultures (e.g. the MV 4–11 cell line) makes the unpaired T-test an unsuccessful approach to detect the significance of the decrease of the phosphocofilin spot brought by LIMK inhibition. The situation is even worse with the 32D- c-kit WT cell line where the inter-experiments variability is such that a 2-fold reduction does not reach the p<0.05 cutoff

### Phosphorylation landscape of cofilin and its modification upon LIMK inhibition

**Fig 4:**
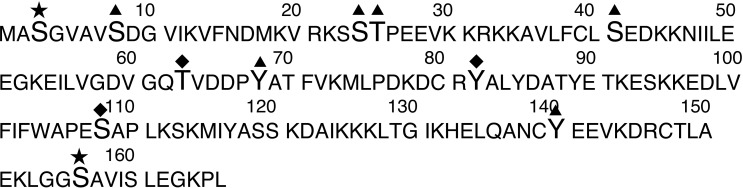
Map of the cofilin phosphosites. The phosphosites are displayed on the sequence of human cofilin. star: already known and described in the present study. triangles: described in the literature but not found in the present study. diamonds: described for the first time in the present study.

In 2D gel electrophoresis, all the spots that bear the same number of phosphate groups comigrate. From the observed pI of the spot (Spot I on Fig 3) and from the blotting data (Spot III on Fig 1), it can be deduced that the phosphorylated spot decreased in intensity upon Pyr1 treatment is doubly modified, e.g. N-terminal acetylated (as most eukaryotic cytosolic proteins are) and mono-phosphorylated. To explore the modification landscape of this cofilin spot, we allowed acetylation and phosphorylation as variable modifications and allowed semi-tryptic peptides and one missed-cleavage during the computer analysis of the mass spectrometry data. This resulted in the identification of several modified peptides, including the expected modified N-terminal peptide (N-terminal methionine removed, alanine-2 acetylated and serine-3 phosphorylated), but also the non-phosphorylated form of this N-terminal acetylated peptide. Several additional phosphorylation sites were pointed out by this analysis, on T63, Y82, S108 and S 156, of which only the latter has been previously described in the literature [41]. These results are displayed in **S1 Table and in Fig 4**. The modified peptides mass spectra are available in the **S2–S7 Figs**.

The detection of other phosphorylation sites prompted us to re-assess the phosphorylation changes induced by the LIMK inhibitor. Indeed, the forms phosphorylated on other sites may induce a basal level in the mono-phosphorylated cofilin spot, thereby artefactually minimizing the decrease induced by LIMK inhibition. To precisely measure the extent of this decrease, we quantitatively compared the signals obtained for each peptide (**S2–S5 Tables)**. As the extraction yield may vary for each spot, e.g. due to its abundance [42] and/or to variability in the spot excision process, we renormalized the peptide signals as the fraction of the total peptide intensities measured for each spot, as described in the methods section. This allowed the calculation of a decrease factor for each of the phosphorylated peptides, which is the combination of this proportion of the phosphopeptide for each spot multiplied by the relative spot abundances measured from the 2D gels. This analysis showed that the reduction in S3 phosphorylation induced by Pyr1 treatment is somewhat higher than detected from the sole quantification of the gel spots. It does not exceed, however, a 5-fold reduction (**Table 4)**.

**Table 4:** Quantitative measurements of the changes in phosphopeptide amounts brought by LIMK inhibition. The data are expressed in fold-changes in the phosphopeptides amounts in the phosphocofilin spot (control/pyr-1 treated). These fold-changes are obtained by the proper combination of the normalized phosphopeptide abundance described in S2–S5 Tables with the spot abundances changes described in Table 3.

	Kasumi	MV4-11	32D c-kit WT	32D c-kit D814V
S3	2.045	4.346	2.744	2.474
T63a	0.901	1.825	1.039	7.105
T63b	1.390	0.820	ND	2.548
T63 sum	1.502	1.422	1.039	5.202
Y82	1.140	1.867	1.991	2.694
S108	1.536	2.429	ND	ND
S156	0.944	1.011	0.872	1.228

ND: modified peptide not detected. Note: as this quantitative analysis process is cumbersome. it has been carried out on one series of phosphocofilin spots (control vs. Pyr-1 treated) for each cell line

## Discussion

ADF/cofilin proteins are considered to be key modulators of actin dynamics in metastasis and invasion of cancer cells [5, 43–46]. The current view of the regulation of the actin severing activity of cofilin is that it is inactivated by its phosphorylation on S3 thanks to LIMK and is reverted to its basal unphosphorylated active state by the phosphatases slingshot 1 (SSH1) and chronophin (see [6, 47] for reviews). Our results indicate that in myeloid cell lines the phosphorylated proportion of cofilin is minor compared to the unphosphorylated, active fraction. This could reflect a difference in actin dynamics of the analyzed cell lines: these cell lines are mainly non adherent and may not need the same amount of actin microfilaments as adherent cell lines. This could also indicate that a part of the cofilin pool is not accessible to phosphorylation. Moreover, we observed that Pyr1 did not completely inhibit S3 cofilin phosphorylation. There are several possible explanations: firstly, depending on the cell line, the membrane permeability could differ, leading to variable intracellular concentrations of Pyr1 and possible incomplete inhibition. Secondly, lower levels of expression or activity of SSH1 or possible subcellular sequestration of phosphorylated cofilin could lead to residual cofilin phosphorylation. Thirdly, it has been shown that other kinases than LIMK are able to phosphorylate cofilin at S3 [48–50]. These Pyr1-insensitive kinases could be responsible of the observed residual cofilin phosphorylation.

A possible similar residual cofilin phosphorylation could also explain why LIMK inhibition did not reduce spontaneous metastasis in animal tumor models [16, 51].

Conversely, the described antitumor effect of LIMK inhibition could involve other subtrates than cofilin. It has been shown that the microtubule cytoskeleton is also impacted by LIMK inhibition [9, 10, 16], by yet unknown mechanisms [3]. There are also evidences that LIMK may bind to and regulate other proteins [15, 52–54], so that other, known or yet unknown substrates may mediate antitumor effects of LIMK inhibition, independently of cofilin.

Finally, our results show that cofilin is phosphorylated at other sites than S3, indicating that cofilin regulation is much more complex than expected. Such other phosphosites have already been described. For instance, it has been previously shown that phosphorylation at Y68 mediates protein degradation [55], while phosphorylation at S23-S24 is also implicated is the modulation of the actin severing function of cofilin and plays a role in cellular degradation [56]. We were not able to detect the Y68 phosphorylation. A plausible explanation is that such a phosphorylation, which impacts protein stability, can be expected to be transient and at a low level, and thus hardly detectable in our experimental conditions. Besides these known sites, we detected new phosphosites, both on tyrosine (Y82), threonine (T63) and serine (S108, S156), indicating that cofilin is a multi-regulated protein. The identification of the kinases responsible of these phosphorylations as well as their functional consequences should shed light on cofilin functions.

## Supporting information

S1 Fig Alignment for the detection of the cofilin spots. A: Silver stained gel image (nonlinear 3–10 pH gradient).
B: India ink staining of a PVDF membrane blotted from an equivalent 2D gel.
C: Immunodetection of the cofilin spots on the membrane shown in B.
D: Immunodetection of the S3 phosphorylated cofilin spots on the membrane shown in B (image cropped on the cofilin region for space reasons).
Blue arrows: spots used to realign the 2D gel image and the stained blot image.
Purple solid lines: alignment of the cofilin spots not phosphorylated on S3.
Red dotted lines: alignment of the S3 phosphorylated cofilin spots.
X: spots detected as cofilin by blotting but not confirmed by mass spectrometry.
Red-circled spots: confirmed cofilin spots (detected by blotting and mass spectrometry.
Blue-circled spots: spots analyzed by mass spectrometry but not identified as cofilin.
Plus sign: Pyr-1 responsive S3 phosphorylated cofilin spot.
Note: the hand-made thick black dotted lines on the B panel were made on the membrane after the immunodetection process to point the major cofilin spots.
(JPG)

S2 Fig Annotated spectrum of the S3 phosphorylated peptide. The annotated MS/MS spectrum of the peptide is shown at the bottom, with the assignment of the fragments on the top of the figure.
(JPG)

S3 Fig Annotated spectrum of the T63 phosphorylated peptide (mouse cofilin). The annotated MS/MS spectrum of the peptide is shown at the bottom, with the assignment of the fragments on the top of the figure.
(JPG)

S4 Fig Annotated spectrum of the T63 phosphorylated peptide (human cofilin). The annotated MS/MS spectrum of the peptide is shown at the bottom, with the assignment of the fragments on the top of the figure.
(JPG)

S5 Fig Annotated spectrum of the Y82 phosphorylated peptide. The annotated MS/MS spectrum of the peptide is shown at the bottom, with the assignment of the fragments on the top of the figure.
(JPG)

S6 Fig Annotated spectrum of the S108 phosphorylated peptide. The annotated MS/MS spectrum of the peptide is shown at the bottom, with the assignment of the fragments on the top of the figure.
(JPG)

S7 Fig Annotated spectrum of the S156 phosphorylated peptide. The annotated MS/MS spectrum of the peptide is shown at the bottom, with the assignment of the fragments on the top of the figure.
(JPG)

S1 Table List of assigned peptides on the modified phosphocofilin spot (Kasumi-1 cell line, spot III). The peptides identified by mass spectrometry are detailed in this table. Modified amino acids appear in lowercase in the peptide sequences, and the modifications are detailed in the corresponding column.
(XLSX)

S2 Table Peptide integration of the phosphocofilin spot, MV 4–11 cell line. The peptides corresponding to all proteins detected in the phosphocofilin are quantified and summed in the control and pyr1-treated conditions. This summed is used to renormalize the intensities of the different peptides, and to calculate the relative peptide abundances at equal total protein mass.
(XLSX)

S3 Table Peptide integration of the phosphocofilin spot, Kasumi-1 cell line. The peptides corresponding to all proteins detected in the phosphocofilin are quantified and summed in the control and pyr1-treated conditions. This summed is used to renormalize the intensities of the different peptides, and to calculate the relative peptide abundances at equal total protein mass.
(XLSX)

S4 Table Peptide integration of the phosphocofilin spot, 32D c-kit WT cell line. The peptides corresponding to all proteins detected in the phosphocofilin are quantified and summed in the control and pyr1-treated conditions. This summed is used to renormalize the intensities of the different peptides, and to calculate the relative peptide abundances at equal total protein mass.
(XLSX)

S5 Table Peptide integration of the phosphocofilin spot, 32D c-kit D814V cell line. The peptides corresponding to all proteins detected in the phosphocofilin are quantified and summed in the control and pyr1-treated conditions. This summed is used to renormalize the intensities of the different peptides, and to calculate the relative peptide abundances at equal total protein mass.
(XLSX)
