# Supplementary figures and images for: A quantitative proteomic analysis of cofilin phosphorylation in myeloid cells and its modulation using the LIM kinase inhibitor Pyr1
Source: PLoS One. 2018 Dec 14;13(12):e0208979. doi: 10.1371/journal.pone.0208979 (PMC6294390; doi:10.1371/journal.pone.0208979)

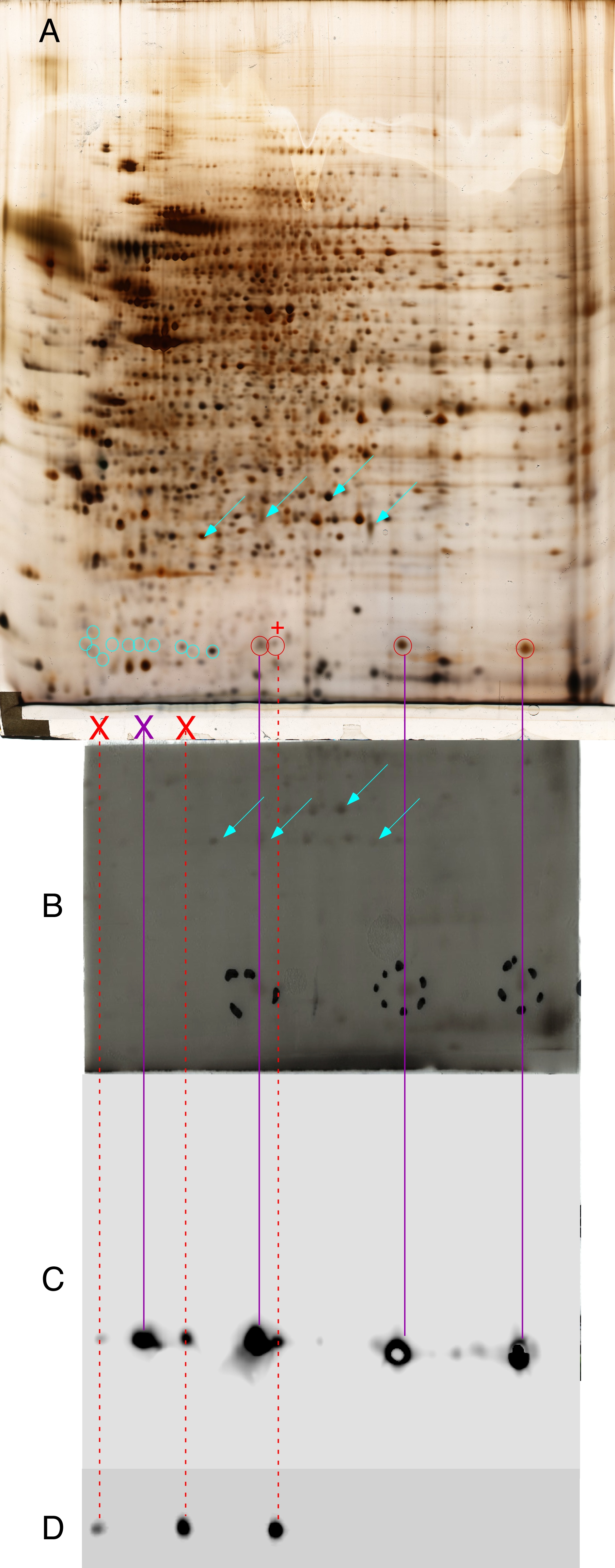

Supplement: S1 Fig — A: Silver stained gel image (nonlinear 3–10 pH gradient). B: India ink staining of a PVDF membrane blotted from an equivalent 2D gel. C: Immunodetection of the cofilin spots on the membrane shown in B. D: Immunodetection of the S3 phosphorylated cofilin spots on the membrane shown in B (image cropped on the cofilin region for space reasons). Blue arrows: spots used to realign the 2D gel image and the stained blot image. Purple solid lines: alignment of the cofilin spots not phosphorylated on S3. Red dotted lines: alignment of the S3 phosphorylated cofilin spots. X: spots detected as cofilin by blotting but not confirmed by mass spectrometry. Red-circled spots: confirmed cofilin spots (detected by blotting and mass spectrometry. Blue-circled spots: spots analyzed by mass spectrometry but not identified as cofilin. Plus sign: Pyr-1 responsive S3 phosphorylated cofilin spot. Note: the hand-made thick black dotted lines on the B panel were made on the membrane after the immunodetection process to point the major cofilin spots. (JPG) [file pone.0208979.s001.jpg]

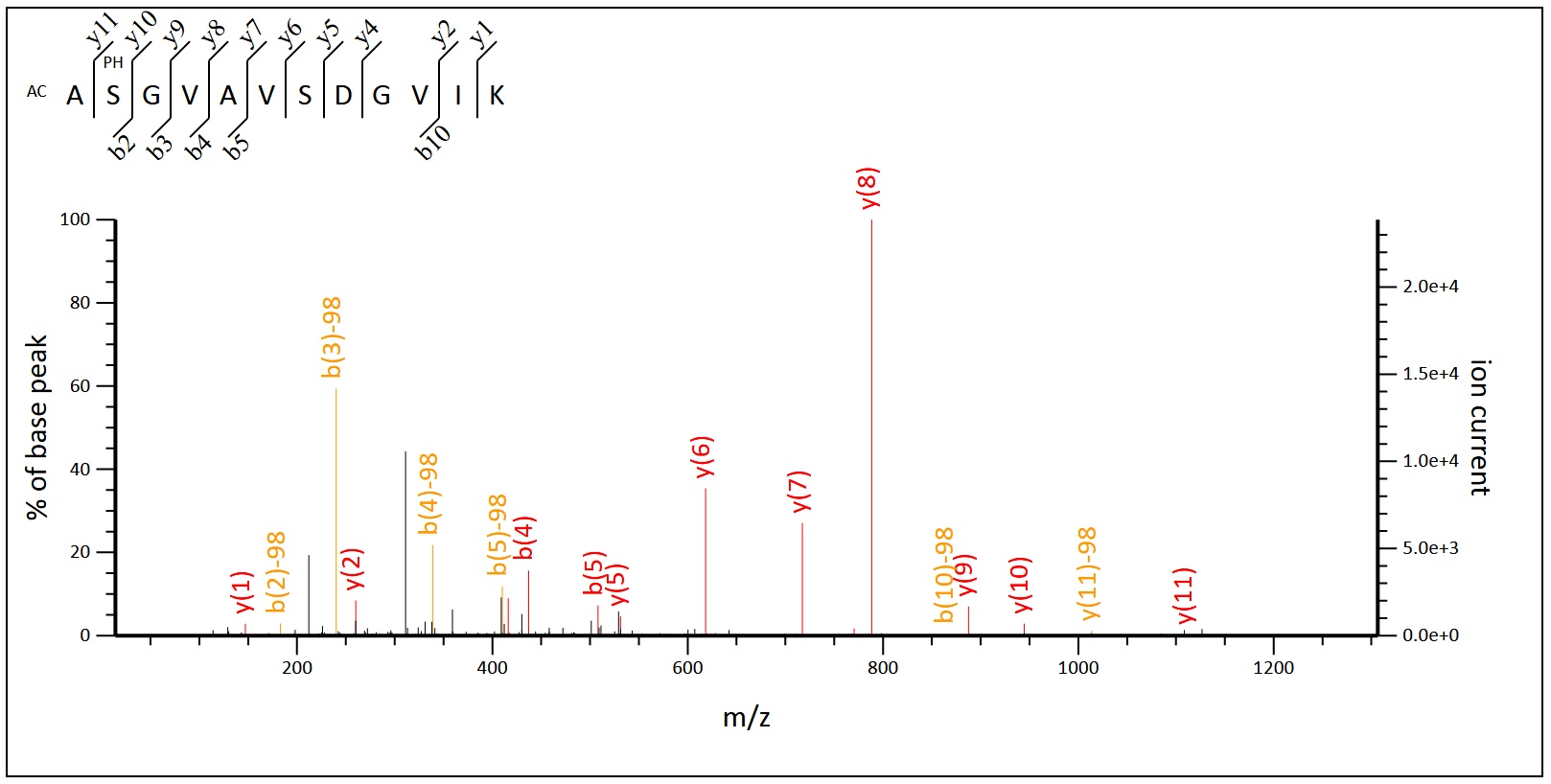

Supplement: S2 Fig — The annotated MS/MS spectrum of the peptide is shown at the bottom, with the assignment of the fragments on the top of the figure. (JPG) [file pone.0208979.s002.jpg]

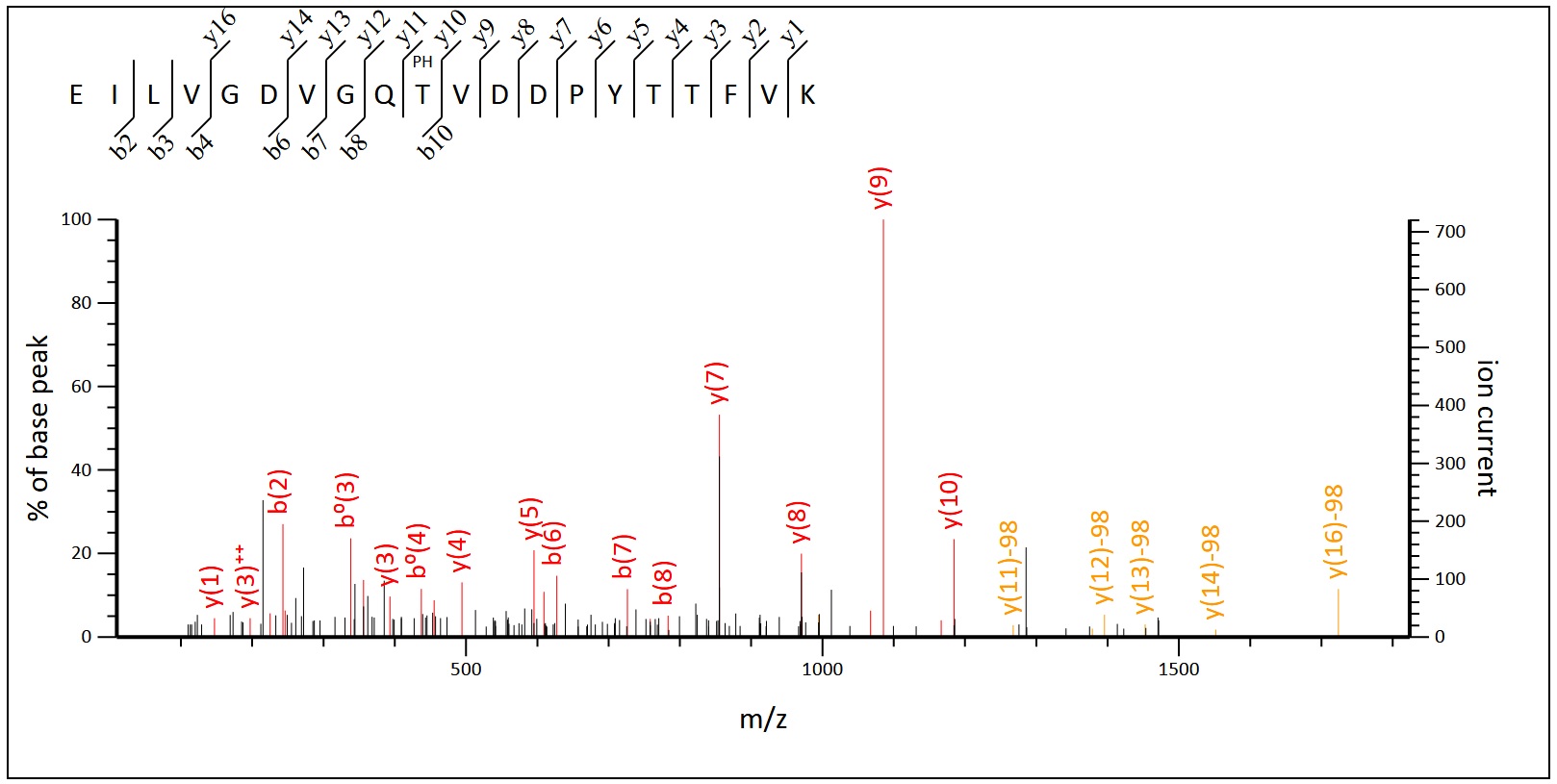

Supplement: S3 Fig — The annotated MS/MS spectrum of the peptide is shown at the bottom, with the assignment of the fragments on the top of the figure. (JPG) [file pone.0208979.s003.jpg]

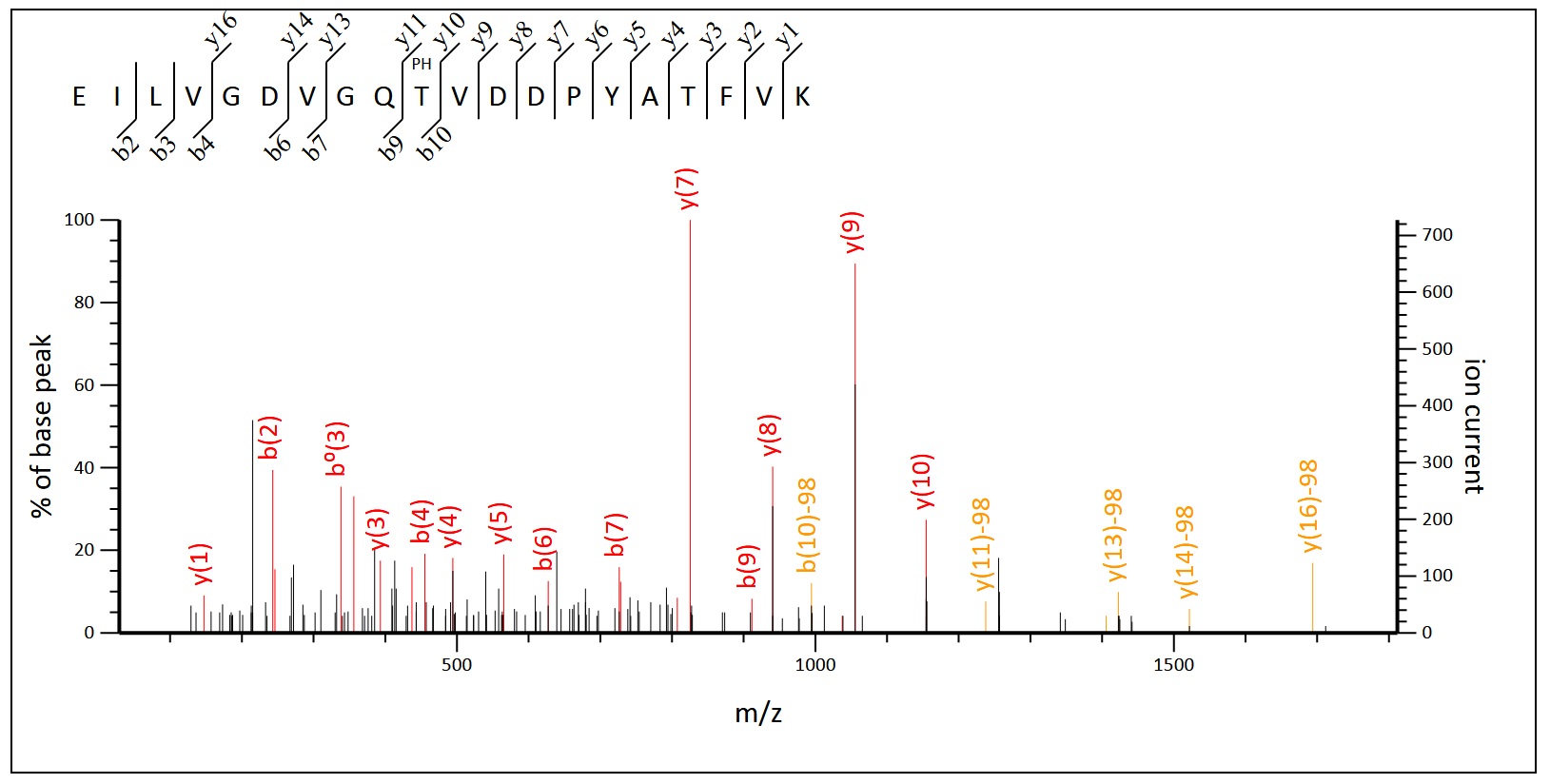

Supplement: S4 Fig — The annotated MS/MS spectrum of the peptide is shown at the bottom, with the assignment of the fragments on the top of the figure. (JPG) [file pone.0208979.s004.jpg]

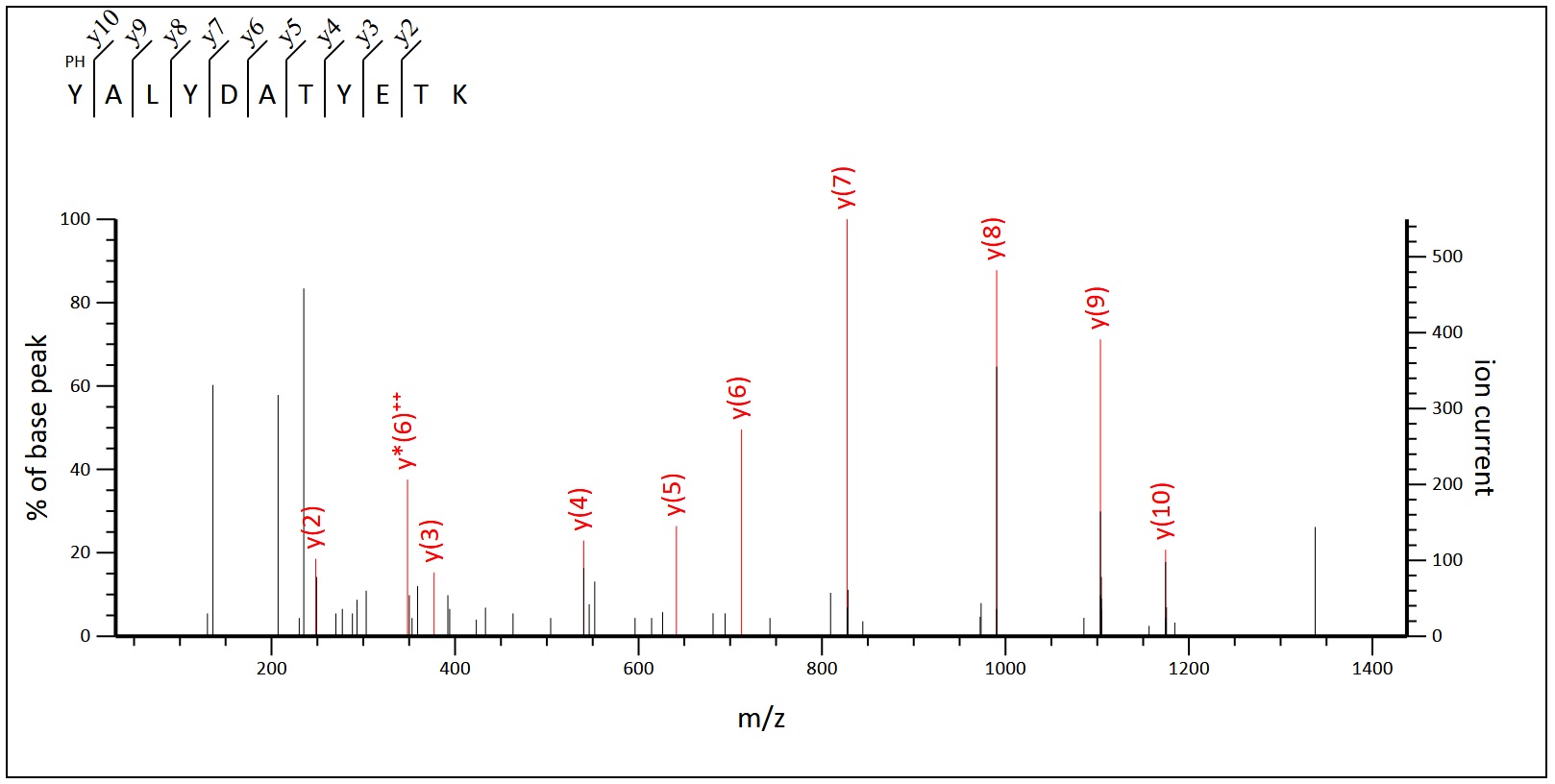

Supplement: S5 Fig — The annotated MS/MS spectrum of the peptide is shown at the bottom, with the assignment of the fragments on the top of the figure. (JPG) [file pone.0208979.s005.jpg]

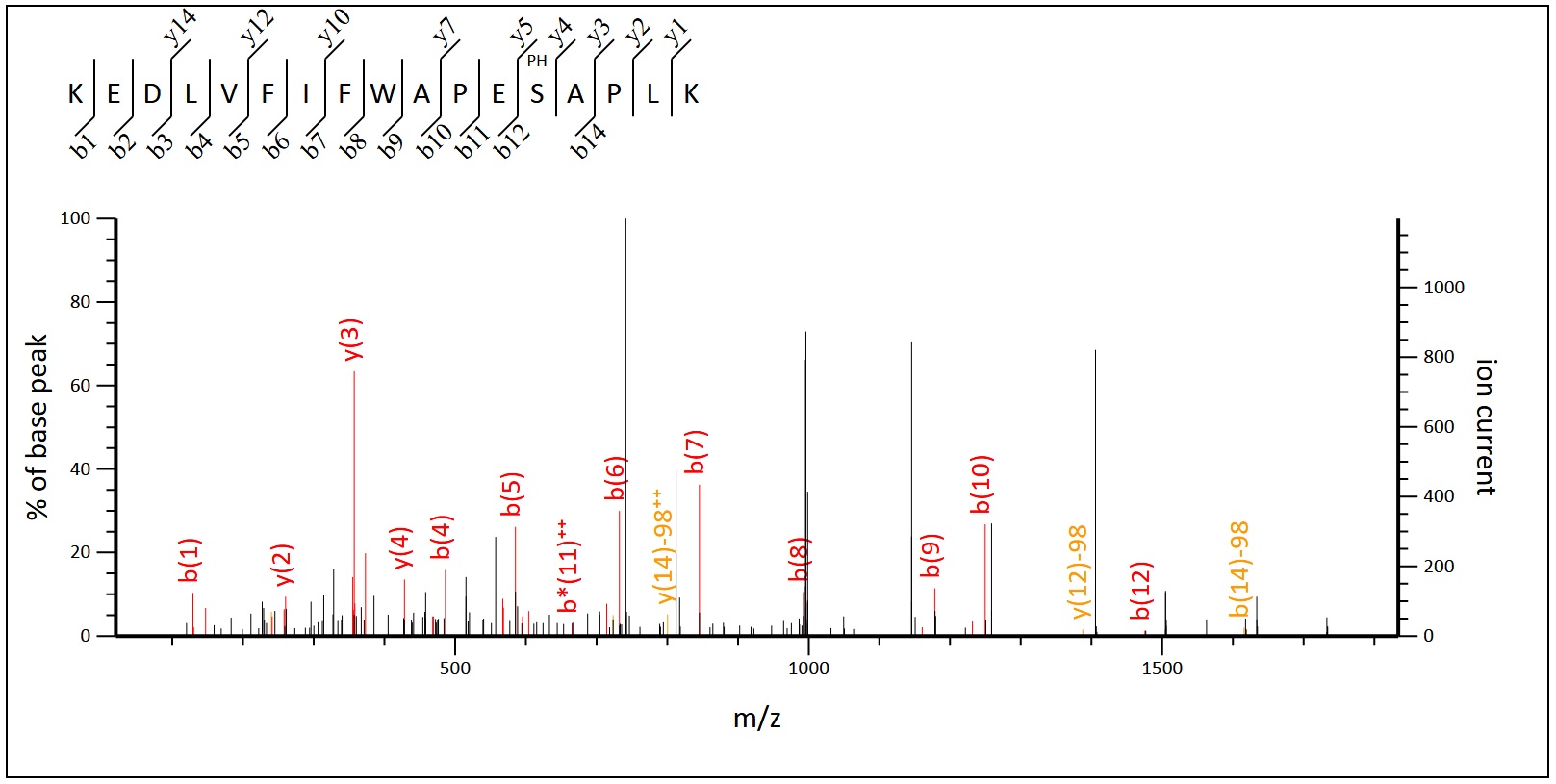

Supplement: S6 Fig — The annotated MS/MS spectrum of the peptide is shown at the bottom, with the assignment of the fragments on the top of the figure. (JPG) [file pone.0208979.s006.jpg]

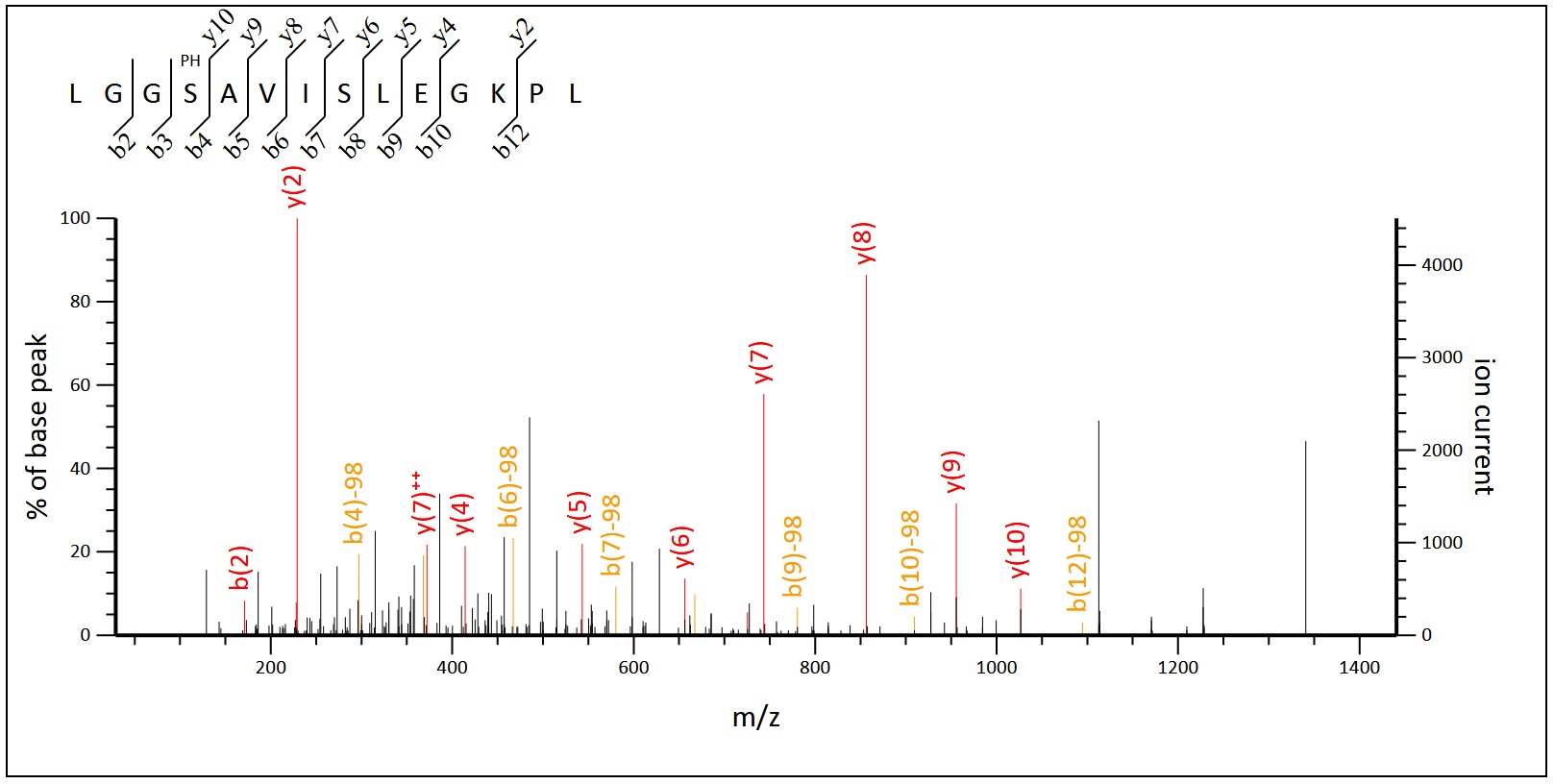

Supplement: S7 Fig — The annotated MS/MS spectrum of the peptide is shown at the bottom, with the assignment of the fragments on the top of the figure. (JPG) [file pone.0208979.s007.jpg]
